# The Temporal Trend of Influenza-Associated Morbidity and the Impact of Early Appearance of Antigenic Drifted Strains in a Southeast Asian Country

**DOI:** 10.1371/journal.pone.0084239

**Published:** 2014-01-08

**Authors:** Ie-Bin Lian, Hong-Dar Isaac Wu, Wan-Tzu Chang, Day-Yu Chao

**Affiliations:** 1 Graduate Institute of Statistics and Information Science, National Changhua University of Education, Changhua, Taiwan; 2 Department of Applied Math, National Chung-Hsing university, Taichung, Taiwan; 3 Institute of Statistics, National Chung-Hsing university, Taichung, Taiwan; 4 Graduate Institute of Microbiology and Public Health, College of Veterinary Medicine, National Chung-Hsing university, Taichung, Taiwan; Fudan University, China

## Abstract

Globally, influenza infection is a major cause of morbidity and mortality in the elderly, who are suggested to be the major target group for trivalent influenza vaccine (TIV) vaccination by World Health Organization. In spite of an increasing trend in vaccine coverage rates in many countries, the effect of vaccination among the elderly in reducing hospitalization and mortality remains controversial. In this study, we conducted a historical cohort study to evaluate the temporal pattern of influenza-associated morbidity among persons older than 64 years over a decade. The temporal patterns of influenza-associated morbidity rates among the elderly older than 64 years indicated that Taiwan's elderly P&I outpatient visits have been decreasing since the beginning of the 1999–2000 influenza season; however, hospitalization has been increasing despite significant increases in vaccine coverage. The propensity score logistic regression model was implemented to evaluate the source of bias and it was found that the TIV-receiving group had a higher propensity score than the non-receiving group (P<0.0001). In order to investigate the major factors affecting the temporal pattern of influenza-associated morbidity, we then used the propensity score as a summary confounder in a multivariate Poisson regression model based on the trimmed data. Our final models suggested that the factors affected the temporal pattern of morbidity differently. The variables including co-morbidity, vaccination rate, influenza virus type A and B isolation rate were associated with increased outpatient visits and hospitalization (p<0.05). In contrast, variables including high propensity score, increased 1°C in temperature, matching vaccine strains of type A/H1N1 and type B were associated with decreased outpatient visits and hospitalization (p<0.05). Finally, we assessed the impact of early appearance of antigenic-drifted strains and concluded that an excess influenza-associated morbidity substantial trends toward higher P&I hospitalization, but not outpatient visits, during the influenza season with early appearance of antigenic-drifted strains.

## Introduction

Globally, influenza infection is a major cause of morbidity and mortality in the elderly[1]. The vaccines currently used against seasonal influenza contain antigens against three influenza strains (A/H1N1, A/H3N2 and B), which are altered yearly to target the strains that are predicted to circulate in the upcoming season (WHO)[2], [3]. The yearly influenza vaccination of at-risk individuals became common practice worldwide after the Second World War. After 2000, more than 40 developed or rapidly developing countries recommended vaccination to prevent influenza and its complications for “high risk” groups, such as the elderly (65 years or older), patients with chronic conditions, and institutionalized populations[4]. Nevertheless, vaccination of the elderly remains controversial. Since there is a gradual decline in immune competence with age, immunogenicity and hence vaccine effectiveness in the elderly are suboptimal[5], [6]. In spite of an increasing trend in vaccine coverage rate, pneumonia and influenza-associated hospitalization and mortality among the elderly have continued to rise in Italy[7], the United States[8] and South Korea[9]. Recent comprehensive literature reviews, based on 75 studies conducted between 1966–2009, revealed that the trivalent inactivated influenza vaccine (TIV) has been proven effective in preventing laboratory-confirmed influenza among healthy elderly[10]. However, there is strikingly limited good-quality evidence and inconsistent results of the effectiveness of influenza vaccination on complications such as pneumonia, hospitalization and influenza-specific and overall mortality, which can only be explained by bias of unknown origin.

Because influenza virus replication has an inherently high mutation rate, the Taiwan Centers for Disease Control (Taiwan-CDC) has coordinated a laboratory-based influenza virological surveillance network (Lab-ISN) starting in 2000 for providing up-to-date information on viral characteristics and activities[11], [12]. In the region of Southeast Asia, there is usually a gap of 1–2 years before an epidemic strain is recommended by the World Health Organization (WHO) to be the vaccine strain[13], [14]. Recent surveys of influenza viruses in Taiwan by hemagglutination (HA) sequence comparisons have indicated a high rate of vaccine mismatch and found that epidemic strains in Taiwan often became the vaccine strains 2–3 years later[12], [15]. Generally, antigenic alterations of local strains that make existing antibody levels in the population no longer protective are considered to be related to disease severity, increased medical utility and vaccine efficacy.

Assessment of influenza vaccine effectiveness is complicated by the degree of match between vaccine and virus, which varies from year to year with mismatches leading to lower vaccine efficacy[16]. Cross-reactivity and avidity of antibody after vaccination might increase through repeated immunization[17]. However, the long-term effect on the reduction of influenza-associated morbidity, defined by medical uses including outpatient visits and hospitalization, has not been evaluated in countries with early appearance of vaccine-mismatched strains. The Taiwan program of targeted free influenza vaccination for people with underlying medical disorders was implemented since 1996 and further expanded in 1998 to include all people older than 64 years. Vaccines are delivered in health care settings by nurses or physicians, or in community settings through public health departments. The vaccination rate gradually increased from 9.9% in 1998 to 49.2% in 2007 with the peak reaching 70% in 2004, which was approximately the target level of 68.0% set by Taiwan-CDC for a 2010 national goal. We conducted a historical cohort study to evaluate the influenza-associated morbidity among persons older than 64 years over a number of epidemic and nonepidemic years in the past decade. The aims of this study were: (1) to evaluate the temporal pattern of influenza-associated morbidity over the last decade, (2) to evaluate the source of bias measured by propensity score and investigate the major factors affecting the temporal pattern of influenza-associated morbidity, (3) to assess the impact of early appearance of antigenic-drifted strains on the excess influenza-associated morbidity.

## Materials and Methods

### Data Sources

The data used in this study were obtained from the National Health Insurance (NHI) Research Database (NHIRD) in Taiwan for the period 2000 to 2009. The NHI program has been implemented in Taiwan since 1995, and the coverage rate was 96% of the whole population in 2000 and over 97% at the end of 2003, at which time more than 21.9 million inhabitants were enrolled. For research purposes, exactly one million residents of Taiwan who were enrolled in NHI in 2005 were randomly selected by the National Health Research Institute in Taiwan, using stratified sampling scheme to ensure the whole population was well-represented. The individual healthcare records for each clinical and hospital visit had been fully digitalized. It should be noted that the rationale for using the NHIRD after 2000 is that, from Jan 1, 2000, according to the rules of the Bureau of NHI, the NHI data were all encoded using the standardized International Classification of Disease, 9 Revision, Clinical Modification (ICD-9-CM).

For each year we acquired the records of those aged 65 or older from NHIRD, and we repeated the process by year from 1999 to 2008. As a result, 60,835 elderly patients (29,462 women and 31,373 men) were used in the analysis for 1999–2000, and the sample size increased yearly to 118,737 (59,721 women and 59,016 men) in the 2008–2009 analysis. Annual influenza periods were marked as beginning on September 1^st^ and concluding on August 31^st^ of the following year.

Data on annual influenza vaccine strains (collected from the WHO), dominant types/subtypes of influenza viruses for winter epidemic seasons, and monthly influenza isolation rates for the 1999–2000 through 2008–2009 influenza seasons were also collected by the Lab-ISN from Taiwan-CDC. Comparisons of the antigenic match between influenza vaccine strains and Taiwan's dominant influenza epidemic strains are summarized in Table S1 and the years with early appearance of antigenic-drifted virus are also indicated.

Vaccination rate data were obtained from three different codings of outpatient visit prescriptions including card sequel number: IC01, ICD-9-CM code: V048, or case type: 92 from the complete NHI claim database in Taiwan for the period 2000 to 2009 since the free vaccination program requires that all the elderly older than 64 use an insurance card to receive vaccination. The results are consistent with the vaccination rate data compiled from local health bureaus by Taiwan-CDC.

### Ethics Statement

For confidentiality and to comply with regulations on personal privacy in Taiwan, all personal identification numbers in NHIRD were encrypted by conversion into scrambled numbers before data processing. Because the database used consists of de-identified secondary data released for research purposes, this principle complies with the Personal Information Protection Act in Taiwan, and this study was exempt from full review by the Institutional Review Board.

### Study Design

We used a retrospective cohort study design to assess the effect of the early appearance of antigenic-drifted strains and the vaccination rate of Taiwan elderly-targeted free influenza vaccination program on influenza-associated morbidity, defined by health care uses including outpatient visits and hospitalization. Although ecological studies do not permit inference to individuals, this design was appropriate for evaluating the impact of a population-level vaccination program on population outcomes rather than the effect of vaccination on individual outcomes. To assess outpatient visits, we included only one service claim per patient per visit per day and excluded claims that were submitted within 14 days of the last claims under the same ICD-9-CM. Emergency room use was not included for analysis because there were few cases annually.

### Definitions

**Figure 1:**
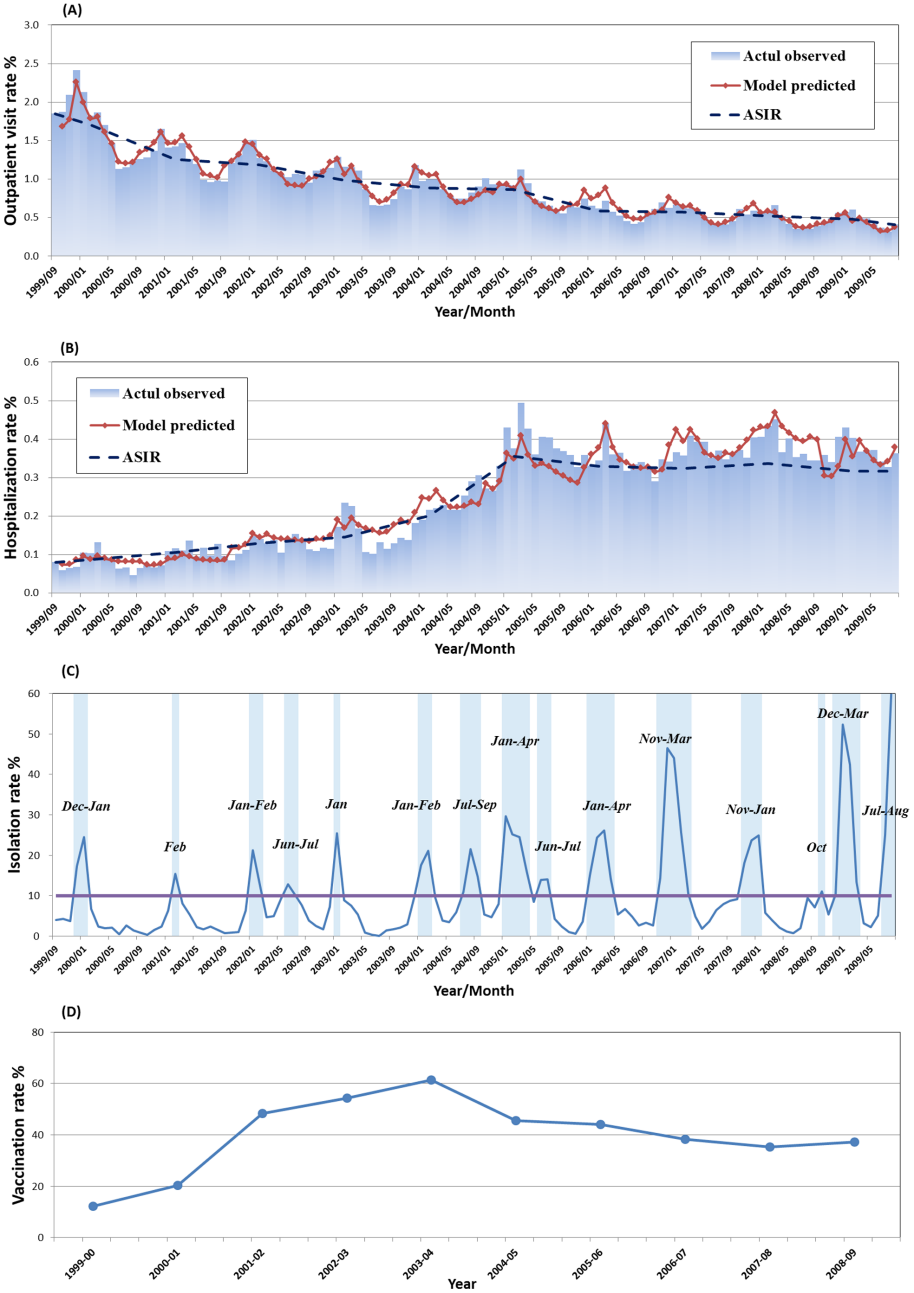
Study event rates among the elderly ≥65 years old over the study period from 1999–2000 to 2008–2009 influenza seasons in Taiwan. Panel A and Panel B represents pneumonia and influenza (P&I)-related outpatient visit and hospitalization rates. The actual observed events (Blue-shaded area), model-predicted events (Red line) and age-standardized events (black dashed line) are expressed as monthly rates per 100. Panel C represents viral surveillance data expressed as the weekly percentage of tests positive for influenza viruses. Horizontal line sets the cutoff of isolation rate ≥10% and the influenza epidemic months are shaded in blue. Panel D represents the annual trivalent influenza vaccine (TIV) vaccination rate among the elderly in percentage. No free TIV vaccination was provided for the 5–19 age group during 1999,9–2007,8.

Influenza-associated morbidities are difficult to quantify because influenza infections are typically not confirmed. Since morbidities coded as influenza are known to underestimate the true burden of influenza, we studied more broadly defined conditions. To better evaluate influenza's disease burden, we included all pneumonia and influenza (P&I, ICD-9-CM: 480–487) from outpatient visits, and hospital discharge diagnoses during the study period, excluding admissions of nonresidents, transfers between institutions, and readmissions within 1 week of discharge. Our case definition did not include positive laboratory confirmation of influenza virus; however, the influenza-associated morbidity peaks during the periods when influenza virus activity increases (Fig 1). Therefore, in order to increase the specificity of the identification of cases hospitalized for influenza-related illness, our influenza-associated outcomes were measured as the difference between observed events and expected baseline events during periods of peak influenza activity (defined below), which reflects the excess number of events that occur because of influenza activity beyond what would be expected from the background (baseline) rates.

Periods of peak influenza activity were defined separately for each year, starting when the weekly percentage of tests positive for influenza was greater than 10% and ending when the percentage fell below that threshold for 2 consecutive weeks.

Underlying diseases were defined as having inpatient or outpatient diagnosis of co-morbidity during the study period. Co-morbidity included existing cardiovascular and other heart diseases (Treatment item #11), chronic bronchitis (Treatment item #10), chronic obstructive pulmonary disease (COPD) (Treatment item #21), bronchiectasis (Treatment item #22), diabetes (Treatment item #01), hypertension (Treatment item #02), and chronic glomerulonephritis (Treatment item #04).

### Propensity Score

When examining the association between y (e.g., influenza-associated morbidity) and x (e.g., vaccination rate), with numerous potential confounders (z's) that are correlated with both x and y, a more plausible way to adjust for the z's is by using a propensity score instead. For dichotomous x, Rosenbaum et al suggested the logit propensity score b(z) =  Log(Pr(x = 1|z)/Pr(x = 0|z)). They had proved that z and x are conditionally independent given b(z)[18]. Since TIV vaccination was preferentially recommended for high-risk frail individuals at many sites including Taiwan, this “confounding by indication” was known a priori that analysis of study outcomes would have to take this source of potential bias into account.

Variables assessed as potential confounders were described as the following. The demographical confounders include: age, gender, geographic region of residence (North/Central/South/East as previously described[19]), urbanization level of residence, and individual socioeconomic status (SES). We used income-related insurance payment amounts as a proxy measure of individual SES at the time of diagnosis. The individuals were classified into three groups: (1) low SES: lower than US$571 per month (New Taiwan Dollar (<NT$20,000); (2) moderate SES: between US$571-1,141 per month (NT$20,000–40,000); and (3) high SES: US$1,142 per month (>NT$40,000) or more. In accordance with Taiwan National Health Research Institute publications[20], urbanization levels in Taiwan are classified into 7 strata, with level 1 referring to the “most urbanized” and level 7 referring to the “least urbanized” communities. Here, levels 6 and 7 were merged into a single group and labeled as level 6 due to the small number of subjects.

We also acquired potential clinical confounders based on clinical information for a one-year period. These clinical confounders included recent transfusion (case type: 05), having tuberculosis (case type: 06), having prescription for chronic diseases (case type: 08–09), having rehabilitation, day-care or long-term care (case type: 61–67), recent infectious diseases including enterobiasis (ICD9: 127.4 or A076), gonorrhea or urogenital gonorrhea (ICD9: 098), syphilis (ICD9: 097 or A060), rubella (ICD9: 056 or A043), measles (ICD9: 055 or A042), human immunodeficiency virus (HIV) infection (case type: V08), varicella or chickenpox-related (ICD-9: 052 or 053), herpes simplex related (ICD-9: 054), herpangina or hand-foot-and-mouth disease related (ICD-9: 074), other viral infection (ICD-9: A049), number of outpatient visits for URI (URI OPD#, ICD-9-CM code: 460-466), recent intestinal disorder (ICD-9-CM code: 558.9 or A549) and clinical record of smoking abstention (case type: 77). Potential confounding variables acquired based on the five-year period preceding the vaccination included the number of outpatient visits, the indicator of having catastrophic illness (defined by NHI with more than 30 different types of diseases, detailed upon request), and the cumulative number of comorbidities of chronic conditions, such as hypertension (treatment item: 02), glomerulonephritis (treatment item: 04), COPD (treatment item: 21), chronic bronchitis (treatment item: 10), diabetes (treatment item: 01), cardiovascular disease (treatment item: 11), and bronchiectasis (treatment item: 22). The ascertainment of the above comorbidities was defined from > = 1 hospital discharge or > = 2 ambulatory visits with a relevant principal or secondary diagnosis code. The indicator variable of recent self-reported flu symptoms was defined as having P&I one-month before and three weeks after the vaccination date (for the vaccinated group) or the first day when the free vaccination was offered (for the unvaccinated group). All variables were acquired by tracking all the ambulatory medical care and inpatient records in the NHI database in the year before the index visit.

To cope with the large number of potential confounders, we used propensity score stratification to create comparable cohorts with different TIV rates. A propensity score logistic regression model was conducted to estimate the probability of receiving TIV versus no TIV. The analyses were carried out annually from 1999 Sep. to 2009 Aug. The logistic regressions were applied to fit the vaccination indicator (1 = yes, 0 = no) on the selected confounders from above, with both the following selection criteria satisfied: (1) is significant in the logistic model, (2) is significantly correlated with the flu indicator (outpatient visit or hospitalization) in chi-square tests.

When fitting the regressions yearly from 1999–2000 to 2008–2009, we found that the selections of variables among the models were quite consistent, with the only exception of Year 1999–2000, which has two variable less than rest of the models (Table S3). The commonly selected variables included geographic region, urbanization, socioeconomic status, numbers of URI, OPD in the previous 6 months, number of comorbidities, number of outpatient visits, having catastrophic illness, and recent intestinal disorder. Based on the results of each model, the propensity score for each person-season was calculated by inverting the estimated logit into the conditional probability for receiving TIV. 
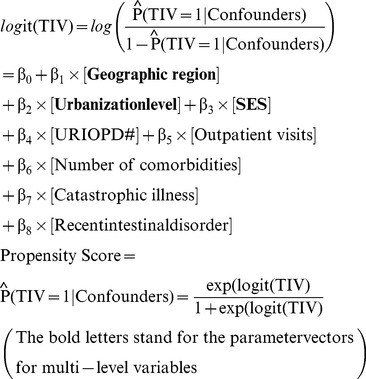
(1)


### Data Analyses and Statistical Tests

We first calculated the crude influenza-associated morbidity rate by using the total population aged ≥65 years as the denominator. Total population was calculated by the population equal to or greater than 65 years old based on the enrollment in NHI in September each year. The age-standardized influenza-associated morbidity rates were calculated based on the age distribution of the world standard population in 2000 from the WHO. Next, we analyzed the influenza-associated morbidity using a three-step procedure. In countries located in sub-tropical regions, summer influenza activity is occasionally observed although winter influenza activity accounts for the major peak. Firstly, in order to select proper annual cyclic or partition function for further multivariate analysis, several Poisson regression models were tried out first to fit the actual influenza activity peaks, including (1) annual periodic cycle functions (i.e., sine-cosine function of seasonal periodicity) and (2) time-trend functions (trisection, quadric-partition, and quinquefid-partition, i.e., divide a 12 month period into 3, 4, or 5 equal parts). The dependent variable was the weekly counts of influenza-associated events, stratified by gender, age and comorbidity combination, and offset by the sample size of each stratum. We then compared the Akaike's information criterion (AIC) and Bayesian information criterion (BIC) among the models to determine the best-fitted one, and chose the corresponding function for further analysis. As a result, all the annual models indicate that quinquefid-partition function has the best fit.

In the second step, the propensity score for each patient was estimated using the model in (1). We then trimmed out the outlier observations defined as those with propensity scores below the lower or above the upper 2.5 tail-percentiles. Stürmer et al suggested that these observations are outside the primary area of the overlap of the propensity score, and may increase residual confounding during analysis[21]. To be used as a stratification variable in the next step, the propensity score was divided into five categories with equal proportions, such that Group 1 has the lowest propensity score and serves as the baseline group.

In the third step, a multivariate Poisson regression approach was used to model the natural logarithm of the expected counts of outpatient visits as well as hospitalization due to P&I. The 10-year data were stratified by the following explanatory variables: Age (65–74, 75–84, 85+), gender, comorbidity indicator (yes vs. no), year indicators (1999.9–2000.8 to 2008.9–2009.8), quinquefid propensity and quinquefid annual cyclic periods (Nov.–Dec., Jan.–Feb., Mar.–May, Jun.–Aug., Sep.–Oct.) which are suggested by the result from Step 1. Besides those stratified variables, the following continuous covariates were also incorporated into the model: annual vaccination rate, average temperature within the period, monthly virus isolation rates for different types/subtypes of influenza viruses (A (H3N2), A (H1N1) and B respectively) and the matching status of different vaccine strains for each type/subtype in each of the studied years. Matching status for each type/subtype of influenza viruses was defined based on the antigenic characteristics of the hemagglutinin gene between the vaccine strain and the dominant wild-type strain of that particular season in Taiwan as published previously[15] and by Taiwan-CDC. The monthly measured data, like temperature and the isolation rates, were averaged over the correspondent period. Furthermore, a scale parameter was added in the Poisson regression to adjust the dispersed distributions. The analysis was implemented by SAS (version 9.3; SAS Institute Inc, Cary, NC). The final model for “influenza-associated morbidity” was devised as follows: 
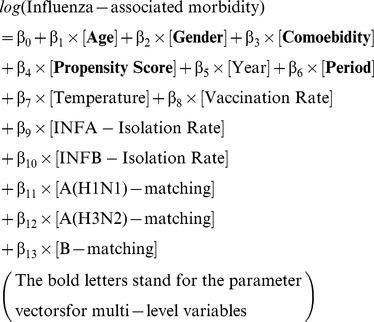
(2)


### Influenza-Associated Excess Morbidities

To evaluate the impact of the early appearance of antigenic-drifted strain on influenza-associated elderly morbidities, excess morbidity (95% confidence interval) was calculated for each winter and year. These excess morbidities including outpatient visits and hospitalization were assessed by calculating the difference between observed data and expected baselines that were derived from the multivariate regression model (Figure 1). After calculating residual morbidity for each month (differences between each winter's monthly observed influenza-associated morbidity and monthly expected value [obtained from our multivariate modeled morbidity baseline]), we replaced negative residuals (e.g. observed values less than the expected value) with zero and summed up excess morbidity for each influenza season. Epidemic (or annual) excess including morbidity during epidemic periods defined by virus isolation rate ≥10% or the whole year were calculated based on each epidemic's (or annual) total excess morbidity divided by the total population aged ≥65 years. The mean values for each epidemic and annual excess morbidity before and after peak vaccination rate were evaluated by independent T-test.

## Results

### Temporal Patterns of Influenza-Associated Morbidity Rates

Temporal patterns of influenza-associated morbidity rates among the elderly older than 64 years indicate that Taiwan's elderly P&I outpatient visits have been decreasing since the beginning of the 1999–2000 influenza season; however, the hospitalization has been increasing despite significant increases in vaccine coverage. As described in Figure 1A, the mean annual P&I outpatient rate, 17.5 per 1000 during the 1999–2000 season, gradually declined to 4.43 per 1000 during the 2008–2009 season. However, the mean annual P&I hospitalization rate during the 1999–2000 season was 1.39 per 1000, which surged to 5.84 per 1000 (p<0.001) after the 2004–2005 season (after the peak coverage rate of implementing a free TIV vaccination program) and remained at a similar rate afterwards (Figure 1B). In addition to this temporal pattern, seasonal cycles were also found for all influenza-associated morbidity rates with peaks consistent with the cyclic peaks of influenza activity based on Lab-ISN as shown in Fig 1C. Noticeably, the yearly vaccination rates among the elderly older than 64 years of age suggested a gradually increasing pattern from 11% during the 1999–2000 season to 60% during the 2003–2004 season; however, the rate has gradually dropped to 40% since then (Fig 1D).

Age-standardized rate also suggested that the outpatient visit rate gradually decreased from 1.71% during the 1999–2000 season to 0.48% during the 2008–2009 season. In contrast, age-standardized hospitalization rate revealed an increase since the 1999–2000 season with 0.09% and peaked at 0.36% during the 2004–2005 season (Fig 1).

### Age-Specific Influenza-Associated Morbidity Rates

**Figure 2:**
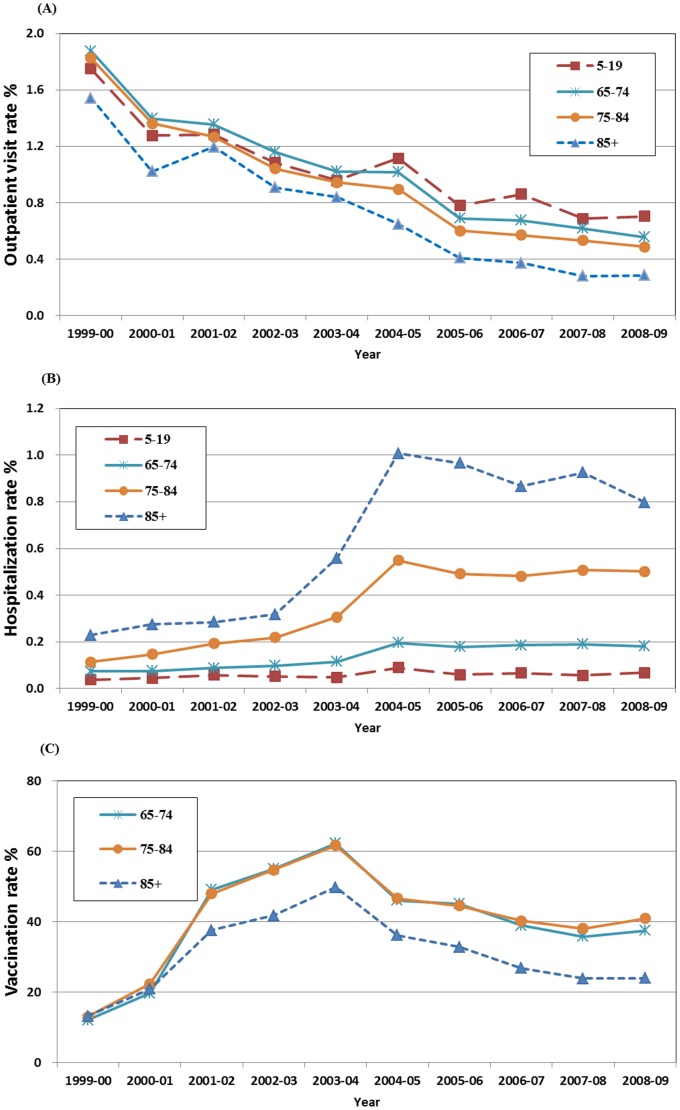
Age-specific event rates over the study period from 1999–2000 to 2008–2009 influenza seasons in Taiwan. Panel A and Panel B represents pneumonia and influenza (P&I)-related outpatient visit and hospitalization rates. Panel C represents the annual trivalent influenza vaccine (TIV) vaccination rate in percentage.

The decreasing patterns of outpatient rates were further analyzed across all age strata. As shown in Fig 2A, the decreasing patterns were consistent throughout the study period among all four age groups (5–19, 65–74, 75–84, and 85+). Interestingly, the outpatient visit rates among the elderly (65–74 and 75–84) were was found to be lower than the rates among the young age group (5–19) after the 2003–2004 season. This was consistent with the increasing annual vaccination rate since 1999–2000 and reached the peak (60% for the 65–74 and 75–84 age group and 50% for the 85+ age group) during the 2003–2004 season. The annual vaccination rate gradually dropped to 20–50% among three age groups of the elderly (Fig 2C). Meanwhile, the increasing patterns of hospitalization rates were also analyzed across all age strata. As shown in Fig 2B, the rates gradually increased since the 1999–2000 season and surged to the peak during the 2004–2005 season, particularly in the both the 85+ and 75–84 age groups. Although the patterns were also consistent throughout the study period, no crossover of the hospitalization rates between the young and elderly age groups was observed.

### Propensity Score Model

The above crude comparison between P&I morbidity rate and TIV vaccination coverage rate among the elderly during the 1999–2009 influenza seasons was confounded by various factors which were unbalanced between groups with and without receiving a TIV vaccination. In order to investigate the source of bias affecting the temporal pattern of influenza-associated morbidity, the logistic regression model was used to predict the probability of receiving TIV vaccination annually. As different people received TIV vaccination each season, the regression model was performed annually and the details of the results after model selection were summarized in Table S2 and S3. We found that the selections of variables among the models were quite consistent when fitting the regressions yearly from 1999–2000 to 2008–2009, with the only exception being the 1999–2000 season, which had two variables that were not selected into the models, including SES and having catastrophic illness.

**Figure 3:**
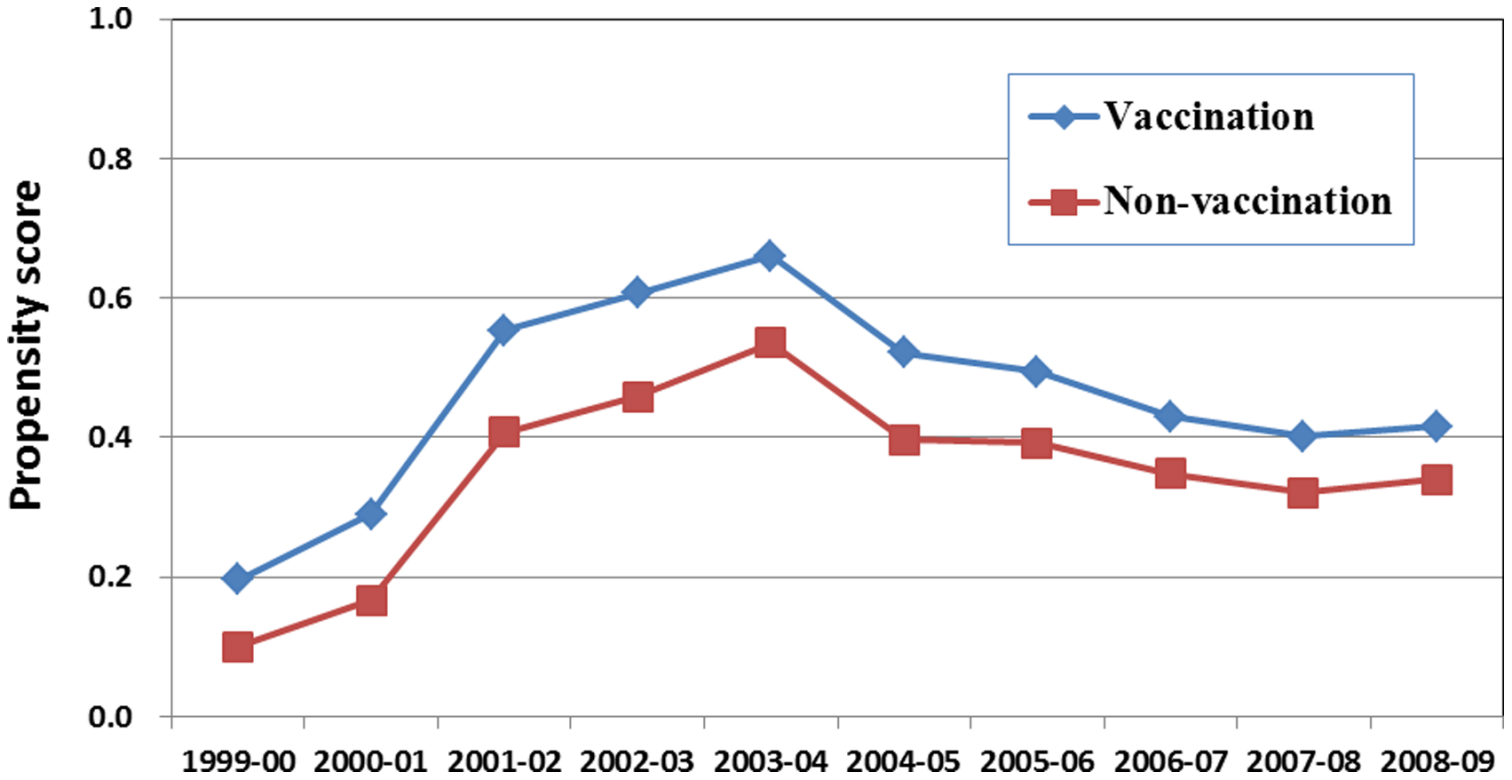
Predicted propensity scores between TIV-receiving and TIV non-receiving elderly over the study period from 1999–2000 to 2008–2009 influenza seasons in Taiwan.

The comparison shown in Table 1 was the summary result with the range of odds ratios (ORs) from both uni-variate and multivariate analysis. There were significant differences in the distribution of geographic region, urbanization level, and SES between the TIV receiving and non-receiving groups. The vaccine-receiving group also had a higher prevalence of pre-existing medical conditions, including numbers of comorbidities (mean OR = 1.23, range:1.12–1.52, p<0.05), numbers of outpatient visits during the past 5 years (mean OR = 1.02, range:1.02–1.03, p<0.05) and recent intestinal disorder (mean OR = 1.56, range:1.40–1.80, p<0.05), than the vaccine non-receiving group. Additionally, the vaccine-receiving group had a lower prevalence of URI OPD# in the previous year (mean OR = 0.89, range:0.85–0.91, p<0.05) than the vaccine non-receiving group. Similar results were observed for multivariate analysis (Table 1). Having catastrophic illness showed a statistically significant difference in prevalence between the vaccine-receiving group and the non-receiving group with an average OR = 0.94 and range between 0.52 and 2.16, with higher prevalence in the vaccine-receiving group in the year before the 2003–2004 season but lower prevalence after the 2003–2004 season. After multivariate analysis, the vaccine-receiving group had lower prevalence in having catastrophic illness than the non-receiving group through all influenza seasons except during the 1999–2000 (not selected by the model) and 2000–2001 with OR = 1.74 (95% CI: 1.58–1.92). In summary, the TIV-receiving group had a higher propensity score than the non-receiving group (P<0.0001) (Fig 3).

**Table 1:** Demographic characteristics and comorbidity medical disorders between TIV-receive and non-receive groups among the elderly ≥65 in years by propensity score logistic regression model.

	OR^b^			Adjust OR^b^		
	Mean	Min	Max	Mean	Min	Max
Geographic region						
North	1.00	1.00	1.00	1.00	1.00	1.00
Central	1.60*	1.22	2.15	1.27*	0.96	1.85
South	1.38*	1.20	1.58	1.15*	1.05	1.38
East	1.28*	1.12	1.63	0.95#	0.83	1.48
Urbanization level						
1 (Most urbanized)	0.61*	0.50	0.75	0.65*	0.53	0.77
2	0.75*	0.66	0.88	0.75*	0.65	0.89
3	0.84#	0.76	1.02	0.86*	0.77	1.11
4	1.00	1.00	1.00	1.00	1.00	1.00
5	1.18*	0.72	1.38	1.15*	0.67	1.35
6 (Least urbanized)	1.17*	1.08	1.47	1.16*	1.09	1.42
Socioeconomic status						
low	1.00	1.00	1.00	1.00	1.00	1.00
moderate	0.84*	0.68	0.96	1.08#	0.89	1.24
high	0.68*	0.56	0.84	0.83#	0.70	0.99
URI OPD# in prior 1 year^a^	0.89*	0.85	0.91	0.84*	0.80	0.86
Outpatient visit frequency	1.02*	1.02	1.03	1.04*	1.03	1.04
Number of Comorbidities	1.23*	1.12	1.52	1.11*	1.01	1.35
Catastrophic Illness	0.94*	0.52	2.16	0.77#	0.40	1.74
Recent Intestinal Disorder	1.56*	1.40	1.80	1.39*	1.12	1.69

^a^ URI OPD: outpatient visits due to upper respiratory infections;

^b^ OR: odds ratio by univariate and multivariate logistic regression;

*p<0.05 throughout all influenza years from 1999–2000 to 2008–2009;

^#^ p<0.05 throughout all influenza years except year 1999–2000.

### Influenza-Associated Morbidity Models

In order to investigate the major factors affecting the temporal pattern of influenza-associated morbidity, we then used the propensity score as a summary confounder in a multivariate Poisson regression model based on the trimmed data, which included the strongest confounders along with the propensity score. As shown in Table 2, two variables associated with the temporal pattern of influenza-associated morbidity were calendar year and month periods. Our analysis found that increased calendar years were associated with decreased outpatient visits with OR = 0.87 (95%CI: 0.86–0.87) but increased hospitalization with OR = 1.13 (95%CI: 1.12–1.13) (p<0.001). Furthermore, the annual influenza epidemic period, which usually occurs between Oct. and Jan. in winter, showed higher influenza outpatient visits with OR 1.22 (95%CI: 1.18–1.26) and 1.14 (95%CI: 1.08–1.19) for month periods Sep.–Oct. and Nov.–Dec., respectively. Conversely, the annual influenza epidemic period showed lower influenza hospitalization risk with OR smaller than 1 compared with the hospitalization rate during the month period of Jun.–Aug.(Table 2), which was usually the baseline period in other studies.

**Table 2:** Primary analysis results of two fitted multivariate Poisson regression models for pneumonia/influenza-associated outpatient visits and hospitalization among the elderly from Sep., 1999 to Aug., 2009.

Parameter	Outpatient		Hospitalization	
	OR	95% CI	OR	95% CI
Age				
65–74	1.00	(reference)	1.00	(reference)
75–84	0.85**	(0.83,0.87)	2.36**	(2.29,2.43)
85+	0.62**	(0.60,0.65)	5.58**	(5.35,5.82)
Sex				
Female	1.00	(reference)	1.00	(reference)
Male	0.84**	(0.83,0.86)	1.75**	(1.70,1.79)
Comorbidity	1.15**	(1.13,1.17)	3.25**	(3.15,3.35)
Propensity Score				
1(Lowest)	1.00	(reference)	1.00	(reference)
2	0.66**	(0.64,0.68)	0.67**	(0.64,0.70)
3	0.78**	(0.76,0.81)	0.65**	(0.62,0.68)
4	1.05**	(1.03,1.08)	0.69**	(0.66,0.72)
5(Highest)	1.74**	(1.70,1.79)	0.86**	(0.83,0.89)
Year	0.87**	(0.86,0.87)	1.13**	(1.12,1.13)
Period				
Jun–Aug	1.00	(reference)	1.00	(reference)
Sep–Oct	1.22**	(1.18,1.26)	0.86**	(0.82,0.90)
Nov–Dec	1.14**	(1.08,1.19)	0.81**	(0.75,0.87)
Jan–Feb	0.95	(0.89,1.01)	0.84**	(0.77,0.92)
Mar–May	1.09**	(1.05,1.14)	0.99	(0.94,1.05)
Temperature	1.00**	(1.00,1.00)	1.01**	(1.01,1.01)
Vaccination Rate	0.96*	(0.96,0.97)	0.98**	(0.97,0.99)
INFA Isolation Rate	1.01**	(1.01,1.01)	1.01**	(1.00,1.01)
INFB Isolation Rate	1.00*	(1.00,1.00)	1.00*	(1.00,1.01)
A/H1N1Vaccine Strain Matching	1.00	(0.97,1.03)	0.76**	(0.73,0.79)
A/H3N2Vaccine Strain Matching	0.99	(0.97,1.02)	1.01	(0.97,1.06)
INF B Vaccine Strain Matching	0.98	(0.96,1.00)	0.95*	(0.92,0.98)

*p<0.05;

**p<0.001.

Our final model also found that increased age was associated with decreased outpatient visits but increased hospitalization. Compared to the age group 65–74, the higher the ages were, the lower the risk of outpatients visits with OR = 0.85 (95%CI: 0.83–0.87) and 0.62 (95%CI: 0.60–0.65) but the higher the risk of hospitalization with OR = 2.36 (95%CI: 2.29–2.43) and 5.58 (95%CI: 5.35–5.82) for the age group 75–84 and ≥85, respectively. Similarly, males were associated with decreased outpatient visits with OR = 0.84 (95%CI: 0.83–0.86) but increased hospitalization with OR = 1.75 (95%CI: 1.70–1.79). Other factors that were statistically significant in the Poisson model included co-morbidity, vaccination rate, influenza virus type A and B isolation rate, high propensity score, increased 1°C in temperature, matching vaccine strains of H1N1 and type B. Among them, co-morbidity, vaccination rate, influenza virus type A and B isolation rate were associated with increased outpatient visits and hospitalization (p<0.05). In contrast, high propensity score, increased 1°C in temperature, matching vaccine strains of type A/H1N1 and type B were associated with decreased outpatient visits and hospitalization with statistical significance (Table 2).

### Influenza-associated excess morbidity in elderly

Examining the records from Table S1, five out of the ten studied years showed early appearance of antigenic-drifted strains including 2003-04 for H3N2, 1999–2000, 2004-05, 2005-06 and 2007-08 for both A subtypes (ie. H1N1 and H3N2), and 2006-07 for H1N1. Four among them appeared after 2004, which was coincided with the declining vaccination rate among the elderly with the peak shown in the 2003-04 season as shown in Fig 1. To evaluate the epidemic excess morbidity in those years with early appearance of antigenic-drifted strain before the declining vaccination rate in 2003 and after, we first reviewed the data on the years with and without early appearance of antigenic-drifted strain before the declining vaccination rate in 2003 (ie. without the effect of declining vaccination rate). Then, we focused on the years with early appearance of antigenic-drifted strain and compared them before 2004 and after 2004 with the declining vaccination rate (ie. to avoid the effect of early appearance of antigenic-drifted strain).

First, when we examined the data on the years before 2004 (ie. before the declining vaccination rate), only the 1999–2000 and 2003-04 seasons showed early appearances of antigenic-drifted strains. The epidemic excess outpatient visits per 1,000 persons during both seasons were 0.48 (95%CI: 0.33–0.62) and 0.50 (95%CI: 0.40–0.60), respectively and, which were no significantly different from those during the 2001-02 season. However, significantly higher hospitalizations were observed in 1999–2000 and 2003-04, where epidemic excess per 1,000 persons were 0.24 (95%CI: 0.22–0.25) and 0.28 (95%CI: 0.23–0.33), respectively, compared to those during the 2000-03 seasons. Similar results were also observed for annual excess with 0.53 (95%CI: 0.49–0.57) and 0.49 per 1,000 (95% CI: 0.42–0.55) during the 1999–2000 and 2003-04 season, respectively, which were also higher than the rest of the years.

Second, when we examined the years with early appearance of antigenic-drifted strain and compared them before 2004 and after 2004, only one season (ie. 2003–2004 season) before 2004 but four seasons (ie. 2004–2005, 2005–2006, 2006–2007, 2007–2008) after 2004 showed early appearance of antigenic-drifted strain. Therefore, we included five seasons for comparison. Further examining the mean value of epidemic and annual excess outpatient visits before 2004 showed 0.50 (95%CI: 0.40–0.60) and 1.11 (95%CI: 0.98–1.25) per 1,000 persons, which was not significantly different from those during the seasons after 2004. Similarly, no statistically significant difference of the epidemic excess of hospitalization between the seasons before 2004 and after 2004 was observed. However, significantly lower annual excess of hospitalization with 0.49 (95%CI: 0.42–0.55) per 1,000 persons during the 2003–2004 season was observed compared to those during the seasons after 2004 (p<0.05).

In summary, substantial trends toward higher P&I hospitalization were observed during the influenza season with early appearance of antigenic-drifted strains.

## Discussion

As worldwide influenza epidemics were considered to be seeded each year by viruses that originated in East or Southeast Asia[22], the early appearance of antigenic-drifted strain in this area posted the threat of human health and vaccination strategy. However, no study has been conducted to understand their impact on influenza-associated morbidity. Each winter, most influenza morbidity and deaths occur among seniors (although they were spared during the 2009 pandemic period)[23]. For nearly a decade, there has been a controversy brewing about the benefits of vaccinating seniors against influenza[4], [24]. More recently analysis of influenza morbidity and mortality time trends in several countries fueled this concern, as these studies saw no decline in flu-related hospitalization and deaths as vaccine coverage rose several fold in seniors[7]–[9]. Our study confirmed that although higher vaccination coverage rate was associated with higher morbidity, there was lower probability of having influenza-associated morbidity among those receiving TIV vaccination who yielded higher propensity scores. Most importantly, the early appearances of antigenic-drifted vaccine-mismatched strains after 2003 were associated with higher morbidity among the elderly in Taiwan. Increased vaccine accessibility for the elderly and timely identification of vaccine-mismatched circulating influenza viruses as well as their antigenic variation is crucial for effective evidence-based public health planning and preparedness.

The first and second aims of our study were to investigate the temporal trend of influenza-associated morbidity including OPD visits and hospitalization and identify source of bias affecting the trend over a decade after the implementation of the free TIV vaccination among the elderly since 1998. The dataset of NHI covering 99% of the population in Taiwan thus avoided the selection bias because of the insurance type. Two secular trends were observed: (1) the outpatient visits showed a declining secular trend but an increase in P&I associated hospitalization from both the results of age direct-adjusted standardized rates and Poisson regression model after adjusting various confounders; (2) the decrease in the morbidity among the elderly (65–74 and 75–84), which was lower than the young age group (5–19) after the 2003–2004 season, was observed only in outpatient visit rates but not in the hospitalization rates. In Taiwan, no free TIV vaccination was provided for the 5–19 age group during the 1999–2006 seasons. However, free TIV vaccination program was expanded to include the 6–7 age group of grade 1–2 in the elementary school during the 2007-08 and 2008-09 seasons. Therefore, the lower outpatient visit among the 65–84 age groups could be due to the effect of TIV vaccination. Previous studies from different populations and countries have demonstrated the benefits of vaccination in the elderly population during a single or 10 consecutive influenza seasons with various vaccine effectiveness depending on the matches between the vaccine and wild-type strain of influenza virus although controversy remained regarding the association between TIV and mortality [4], [10], [25]–[29]. Our study also suggested profound effects of TIV in reducing the outpatient visits among the elderly secularly. In contrast, the increasing trend of hospitalization rate in this study was consistent with previous publication[30]. Although the possibility of insurance cost to encourage hospitalization has been considered, expenditures for outpatient care exceeded inpatient care expenditures by a 2∶1 ratio after implementation of NHI in Taiwan based on the study by Wen et al [31] and the most likely reason might be related to the frailty among the elderly because of the deleterious immunological function[32], [33]. Based on our Poisson regression model, the oldest age group (older than 84 years) had the highest risk of hospitalization because of influenza compared to the age group of 65–74 with OR = 5.58 (95%CI: 5.35–5.82). Similarly, the elderly with co-morbidity was statistically significantly associated with hospitalization with OR = 3.25 (95%CI: 3.15–3.35). On the other hand, it was also plausible that other confounding variables resulted in ecological fallacy, such as “healthy-vaccinee effect”[34], the geographic variations and disparities in vaccine delivery and vaccination rates[35], the aging of the population[33], or the increasing numbers of elderly persons with high-risk conditions for whom the risk of dying increases exponentially[36]. Our study used the propensity score to mitigate the problem of bias. Furthermore, methodological research has shown that calculating propensity scores does provide the ability to identify outliers outside the central distribution of the other group, and trimming improves the validity by restricting the study to comparable observations[21]. Our study found that although higher vaccination rates were associated with higher morbidity in hospitalization, there was a lower probability of having influenza-associated morbidity among those receiving TIV vaccination that yielded higher propensity score. This could be due to patients with higher propensity scores tending to be more vulnerable to influenza and having more contact with providers who encourage vaccination. A previous study suggested that incomplete matched influenza vaccine still provide protection in frail elderly[37]. Therefore, our study did not only identify the important sources of bias when analyzing the secular trend of P&I-associated morbidity, but also strengthened the necessity of TIV vaccination among the elderly including the frail persons.

Several observational studies have noticed that morbidity and mortality were relatively low in vaccinees even before the start of flu seasons and suggested the differences in mortality among vaccinated vs unvaccinated is attributable to selection bias[38], [39]. They used the time period during the influenza season and outside influenza season, which are referred to as difference-in-differences approach, to trace the trajectory of the bias over time[40]. However, this approach was difficult to implement in the sub-tropical countries in Southeast Asia where the influenza season was not easily defined and summer influenza epidemics were common as shown in Fig 1 and Table S1. Additionally, the specificity of disease classification based on ICD-9-CM usually presented a problem in many observational studies[30], [41]. In our study, we included the virus isolation rates of type A and B of influenza virus to increase the model fitting and the temporal trend based on the predicted model results demonstrated comparable peaks between the observed and predicted values, even during the peak of summer flu as shown in Fig 1. Interestingly, we found that our data suggested using months Jun-Aug as a baseline, the morbidity of the other months was lower only in outpatient visits but hospitalization in contrast was higher. Our study suggested that there is relative difficulty in conducting a vaccine effectiveness study using observational data in sub-tropical countries in Southeast Asia and more sophisticated epidemiological methods are required in the future.

The third aim of our study, which is also the first study to our knowledge, was to investigate the impact of early appearance of vaccine-mismatched strains on influenza-associated morbidity. Two findings were presented here: (1) the mismatches between the vaccine and wild-type strains, particularly type A H1N1 and type B, were associated with the P&I-associated hospitalization with OR = 0.76 (95%CI: 0.73–0.79) and 0.95 (95%CI: 0.92–0.98), respectively, but not with outpatient visits (Table 2); (2) by either examining the peak excess morbidity in those years with early appearance of antigenic-drifted strain before the declining vaccination rate in 2003 or comparing them before and after 2004, higher P&I hospitalizations were observed during the influenza seasons with early appearance of antigenic-drifted strains (Table 3). TIVs are most effective when there is a good match between circulating viruses and vaccine strains; protection may also be substantial, though sometimes lower, during years with a poor match among healthy adults, elderly at high risk or institutionalized[25]–[27]. More importantly, the early appearance of vaccine-mismatched strains of influenza virus presented a large medical burden of health in the elderly.

**Table 3:** Epidemic and annual excess influenza-associated morbidity rates among the elderly (per 1,000).

	Vaccine matching	early appearance^a^	Peak vaccine coverage in 2004^b^	Epidemic^c^ excess (95% CI)	Annual^d^ excess (95% CI)	
A. Outpatient						
1999–2000	H1, H3 mismatch	Yes	before	0.48 (0.33,0.62)	1.94 (1.69,2.19)	
2000–2001	B mismatch	No	before	0.08 (0.04,0.12)	0.99 (0.85,1.12)	
2001–2002	B mismatch	No	before	0.52 (0.41,0.63)	1.32 (1.14,1.49)	
2002–2003	No mismatch	No	before	0.11 (0.06,0.15)	1.08 (0.93,1.23)	
2003–2004	H3, B mismatch	Yes	before	0.50 (0.40,0.60)	1.11 (0.98,1.25)	
2004–2005	H1, H3, B mismatch	Yes	after	0.70 (0.59,0.80)	1.31 (1.16,1.47)	
2005–2006	H1, H3 mismatch	Yes	after	0.14 (0.10,0.18)	0.54 (0.45,0.63)	
2006–2007	H1 mismatch	Yes	after	0.38 (0.30,0.46)	0.87 (0.75,0.98)	
2007–2008	H1, H3, B mismatch	Yes	after	0.16 (0.11,0.21)	0.82 (0.70,0.93)	
2008–2009	No mismatch	No	after	0.83 (0.70,0.96)	1.26 (1.11,1.41)	
B. Hospitalization						
1999–2000	H1, H3 mismatch	Yes	before	0.24 (0.22,0.25)	0.53 (0.49,0.57)	
2000–2001	B mismatch	No	before	0.03 (0.02,0.05)	0.36 (0.31,0.40)	
2001–2002	B mismatch	No	before	0.12 (0.09,0.15)	0.30 (0.25,0.34)	
2002–2003	No mismatch	No	before	0.04 (0.02,0.06)	0.30 (0.24,0.35)	
2003–2004	H3, B mismatch	Yes	before	0.28 (0.23,0.33)	0.49 (0.42,0.55)	
2004–2005	H1, H3, B mismatch	Yes	after	0.59 (0.50,0.67)	0.99 (0.88,1.10)	
2005–2006	H1, H3 mismatch	Yes	after	0.21 (0.17,0.25)	0.74 (0.66,0.83)	
2006–2007	H1 mismatch	Yes	after	0.26 (0.21,0.31)	0.70 (0.60,0.79)	
2007–2008	H1, H3, B mismatch	Yes	after	0.15 (0.11,0.20)	0.65 (0.56,0.75)	
2008–2009	No mismatch	No	after	0.55 (0.47,0.64)	0.85 (0.75,0.96)	

^a^ Early appearance of antigenic-drifted strains: follow the results from Table S1.

^b^ Peak vaccine coverage in 2004: “Before” and “after” indicated influenza season before or after the peak vaccination coverage rate during 2003–2004 as shown in Fig 1.

^c^ Epidemic: isolation Rate >10%;

^d^ Annual: from September to the following August.

Our data and findings have limitations. First, potential missing data on flu shots given without registry on the insurance card was possible. However, it won't largely affect the results since the vaccination rates derived from NHIRD during the study period were consistent with the governmental registry data organized by Taiwan-CDC (data not shown). Second, we only used variables obtained from the NHIRD for propensity score calculation to adjust the care-seeking behavior, which may vary across social-cultural settings and practice settings. Third, physical functionality is an important confounder [33], [42] and we only used propensity score as a proxy to measure the functionality. Fourth, smoking status was not available. It is well known that individuals who receive influenza vaccinations are less likely to smoke than individual who do not, and that smokers are more likely to get influenza[39], [43]. Fifth, other unknown variables including the contact patterns among the elderly, anxiety over side effects, disease threat perception, and benefits education provided were not available.

In summary, our study investigated the temporal trend of influenza-associated morbidity including OPD visits and hospitalization, and source of bias to correlate the vaccination rates and morbidity data over a decade after the implementation of the free TIV vaccination among the elderly since 1998. Most importantly, the early appearance of antigenic-drifted strains of influenza viruses was correlated with the increased risk of P&I-associated hospitalization among the elderly. A vaccine efficacy/effectiveness study will be important to confirm this result in the future. Achieving optimal success in preventing and controlling influenza among the elderly may require more immunogenic vaccines which are still under development and have not yet been approved for use. Vaccination of school-aged children has been suggested to be associated with lower morbidity and mortality among the elderly in the community[44]–[47]. However, these studies are not conclusive and additional research is needed to define the benefits among the elderly. In the meantime, vaccination rates of elderly persons remain stagnant and well below 50%. The health care providers and policymakers should renew efforts to improve the delivery of current influenza vaccines to the elderly, particularly the group with high propensity score. Additionally, the inclusion of local strains which appeared antigenic-drifted based on the surveillance system in the composition of TIV among the elderly warrants careful consideration particularly in East or Southeast Asian countries.

## Supporting Information

Table S1 Annual comparison between vaccine and circulating wild-type dominant strains of human A/H1N1, A/H3N2 and B viruses isolated in Taiwan.
(DOCX)

Table S2 Annual crude analysis results of uni-variate propensity logistic regression models from 1999–2000 to 2008–2009 influenza seasons.
(DOCX)

Table S3 Annual adjusted analysis results of multivariate propensity logistic regression models from 1999–2000 to 2008–2009 influenza seasons.
(DOCX)
