# Supplementary material for: The Temporal Trend of Influenza-Associated Morbidity and the Impact of Early Appearance of Antigenic Drifted Strains in a Southeast Asian Country
Source: PLoS One. 2014 Jan 8;9(1):e84239. doi: 10.1371/journal.pone.0084239 (PMC3885564; doi:10.1371/journal.pone.0084239)
Supplement: Table S1 — Annual comparison between vaccine and circulating wild-type dominant strains of human A/H1N1, A/H3N2 and B viruses isolated in Taiwan. (DOCX) [file pone.0084239.s001.docx]

Table S1. Annual comparison between vaccine and circulating wild-type dominant strains of human A/H1N1, A/H3N2 and B viruses isolated in Taiwan

| Season | Type | WHO recommended vaccine strains | Epidemic strains in Taiwan | Antigenic match§ | Epidemic period (isolation rate >=10%) | Dominant type |
| --- | --- | --- | --- | --- | --- | --- |
| 1999-2000 |  |  |  |  |  |  |
|  | A/H1N1  A/H3N2  B | Beijing/265/95-like  Sydney/05/97-like  Shangdong/07/97-like or Beijing/184/93-like(Yamagata lineage) | New Caledonia/20/99*  A/Beijing/262/95  Sydney/05/97  Panama/2007/99*  Shangdong/07/97 and Beijing/184/93 | **-**  **+**  **+**  **-**  **+**  **+** | Dec.-Jan. | A |
| 2000-2001 |  |  |  |  |  |  |
|  | A/H1N1  A/H3N2  B | Caledonia/20/99-like  Moscow/10/99-like  Beijing/184/93-like(Yamagata lineage) | Caledonia/20/99-like  Moscow/10/99-like  Sichuan/379/99 | **+**  **+**  **-** | Feb. | B |
| 2001-2002 |  |  |  |  |  |  |
|  | A/H1N1  A/H3N2  B | New Caledonia/20/99-like  Moscow/10/99-like  Sichuan/379/99-like(Yamagata lineage) | New Caledonia/20/99-like  Moscow/10/99-like  Hong Kong/330/2001 | **+**  **+**  **-** | Jan.-Feb  Jun.-Jul. | A |
| 2002-2003 |  |  |  |  |  |  |
|  | A/H1N1  A/H3N2  B | New Caledonia/20/99-like  Moscow/10/99-like  Hong Kong/330/2001-like(Victoria lineage) | New Caledonia/20/99-like  Moscow/10/99-like  Hong Kong/330/2001 | **+**  **+**  **+** | Jan. | A |
| 2003-2004 |  |  |  |  |  |  |
|  | A/H1N1  A/H3N2  B | New Caledonia/20/99-like  Moscow/10/99-like  Hong Kong/330/2001-like(Victoria lineage) | New Caledonia/20/99-like  Moscow/10/99-like  Fujian/411/2002*  Sichuan/379/99 | **+**  **+**  **-**  **-** | Dec.-Feb.  Jul.-Aug. | A |
| 2004-2005 |  |  |  |  |  |  |
|  | A/H1N1  A/H3N2  B | New Caledonia/20/99-like  Fujian/411/2002-like  Shanghai/361/2002-like(Yamagata lineage) | New Caledonia/20/99-like  Solomon Island/3/06*  California/7/2004*  Shanghai/361/2002 and Malaysia/2506/2004 | **+**  **-**  **-**  **+**  **-** | Sep.  Jan.-Jul. | B/A |
| 2005-2006 |  |  |  |  |  |  |
|  | A/H1N1  A/H3N2  B | New Caledonia/20/99-like  California/7/2004-like  Shanghai/361/2002-like(Yamagata lineage) | New Caledonia/20/99 Solomon Island/3/06*  Wisconsin/67/05*  Not available | **+**  **-**  **-**  **+** | Jan.-Apr. | A |
| 2006-2007 |  |  |  |  |  |  |
|  | A/H1N1  A/H3N2  B | New Caledonia/20/99-like  Wisconsin/67/05 or Hiroshima/52/2005  Malaysia/2506/2004(Victoria lineage) | New Caledonia/20/99-like  Solomon Island/3/06*  Wisconsin/67/2005-like  Malaysia/2506/2004-like | **+**  **-**  **+**  **+** | Nov.-Mar. | B/A |
| 2007-2008 |  |  |  |  |  |  |
|  | A/H1N1  A/H3N2  B | Solomon Island/3/06  Wisconsin/67/2005 or Hiroshima/52/2005  Malaysia/2506/2004 | Brisbane/59/2007-like*  Brisbane/10/2007-like*  Florida/4/2006-like | **-**  **-**  **-** | Nov.-Jan. | A |
| 2008-2009 |  |  |  |  |  |  |
|  | A/H1N1  A/H3N2  B | Brisbane/59/2007-like  Brisbane/10/2007-like  Florida/4/2006-like | Brisbane/59/2007-like  Brisbane/10/2007-like  Florida/4/2006-like | **+**  **+**  **+** | Nov.-Feb. | A |

*Epidemic strain circulated in Taiwan earlier than in other countries and prior to WHO vaccine recommendation

§ "+" and "-" indicated antigenic match and mis-match between the vaccine and wild-type circulating strain.
