# Supplementary material for: The Temporal Trend of Influenza-Associated Morbidity and the Impact of Early Appearance of Antigenic Drifted Strains in a Southeast Asian Country
Source: PLoS One. 2014 Jan 8;9(1):e84239. doi: 10.1371/journal.pone.0084239 (PMC3885564; doi:10.1371/journal.pone.0084239)
Supplement: Table S2 — Annual crude analysis results of uni-variate propensity logistic regression models from 1999–2000 to 2008–2009 influenza seasons. (DOCX) [file pone.0084239.s002.docx]

Table S2. Annual crude analysis results of uni-variate propensity logistic regression models from 1999-2000 to 2008-2009 influenza seasons
